# Severe Acute Respiratory Syndrome Coronavirus 2 Shedding by Travelers, Vietnam, 2020

**DOI:** 10.3201/eid2607.200591

**Published:** 2020-07

**Authors:** Thi Quynh Mai Le, Taichiro Takemura, Meng Ling Moi, Takeshi Nabeshima, Le Khanh Hang Nguyen, Vu Mai Phuong Hoang, Thi Hong Trang Ung, Thi Thanh Le, Vu Son Nguyen, Hong Quynh Anh Pham, Tran Nhu Duong, Hai Tuan Nguyen, Duy Nghia Ngu, Cong Khanh Nguyen, Kouichi Morita, Futoshi Hasebe, Duc Anh Dang

**Affiliations:** National Institute of Hygiene and Epidemiology, Hanoi, Vietnam (T.Q.M. Le, L.K.H. Nguyen, V.M.P. Hoang, T.H.T. Ung, T.T. Le, V.S. Nguyen, H.Q.A. Pham, T.N. Duong, H.T. Nguyen, D.N. Nguyen, C.K. Nguyen, D.A. Dang);; World Health Organization Collaborating Center for Reference and Research on Tropical and Emerging Virus Diseases, Institute of Tropical Medicine, Nagasaki University, Nagasaki, Japan (T. Takemura, M.L. Moi, T. Nabeshima, K. Morita, F. Hasebe)

**Keywords:** presymptomatic, 2019 novel coronavirus disease, COVID-19, severe acute respiratory syndrome coronavirus 2, SARS-CoV-2, viruses, respiratory infections, zoonoses, travelers, Vietnam

## Abstract

We analyzed 2 clusters of 12 patients in Vietnam with severe acute respiratory syndrome coronavirus 2 infection during January–February 2020. Analysis indicated virus transmission from a traveler from China. One asymptomatic patient demonstrated virus shedding, indicating potential virus transmission in the absence of clinical signs and symptoms.

During the past 2 months, emergence of 2019 novel coronavirus disease (COVID-19) has caused global public health concern (*1*,*2*). In light of the rapid global expansion of the disease, we performed a detailed epidemiologic and clinical assessment to determine the transmission patterns of the severe acute respiratory syndrome coronavirus 2 (SARS-CoV-2) outside China. As of March 6, 2020, imported SARS-CoV-2 infections have been identified in Vietnam; 17 cases (none severe) have been confirmed. Patients were 3 months to 65 years of age. Although transmission of SARS-CoV-2 may occur within days after illness onset, data on the early viremia kinetics in travelers are limited. We describe the virus shedding patterns in a cluster of travelers and in a cluster of patients who had close contact with the travelers. 

On November 15, 2019, eight employees from a company in northern Vietnam (7 from Vinh Phuc Province, 1 from Thanh Hoa Province) were sent to Wuhan, China, for technical training for ≈2 months. On January 17, 2020, they returned to Vietnam via a flight from Wuhan to Guangzhou, China, followed by a flight from Guangzhou to Hanoi, Vietnam. During January 21–27, 2020, fever and cough developed in 6 of those travelers. Real-time PCR confirmed SARS-CoV-2 infection in all 6 travelers. Virus isolation and next-generation sequencing were performed for samples that were positive for viral RNA. The remaining 2 employees that had been on the same flight were quarantined, but real-time PCR indicated that they were negative for viral RNA. Patients were hospitalized at the National Hospital of Tropical Diseases, Thanh Hoa Provincial Hospital, and Tam Dao District Hospitals in Vinh Phuc Province, where they were closely monitored in isolated wards and followed up. Patient throat swab samples were sent to the Institute of Hygiene and Epidemiology, Hanoi, Vietnam, for laboratory diagnosis. The patients provided consent to have their details shared.

Among those tested for viral RNA by SARS-CoV–specific reverse transcription PCR, results were negative for 155 persons who had been in close contact with the 6 SARS-CoV–positive travelers and 1,092 persons exhibiting clinical signs, including cough and fever (*3*). Those with positive results were 6 of the persons sent to Wuhan for training (cluster 1), another 5 (cluster 2) who had been in close contact with a patient from cluster 1, and 1 patient in cluster 2 (patient 12) who had been in close contact with 2 other patients from cluster 2 (Table). We monitored SARS-CoV-2 viremia in 30 throat swab specimens obtained from the 12 patients in hospitals throughout Vietnam (3 male and 9 female; average age 31.2 years [range 3 months–55 years]).

**Table:** Characteristics of patients within 2 clusters of severe acute respiratory syndrome coronavirus 2 infection in Hanoi, Vietnam, December 2019–February 2020*

Cluster, contact period, patient no. (relationship)	Age/sex	Possible incubation period, d†	Disease onset	Symptom onset to sample collection, d				Virus shedding period, d‡
					Virus genome levels, Ct			
					E gene	*RdRp* gene	N gene	
Cluster 1: Travelers returning from Wuhan								
2019 Nov 15–2020 Jan 17								
1	25 y/F	7	Jan 24	0	25.1	28.0	28.3	7
2	29 y/M	4	Jan 21	5	35.3	38.6	38.0	6
3	23 y/F	8	Jan 25	2	32.0	34.7	34.6	6
4	29 y/F	12	Jan 29	4	30.2	37.1	27.7	7
5	30 y/M	9	Jan 26	6	28.4	>40.0	30.0	1
6	30 y/F	14	Jan 31	0	33.2	>40.0	35.1	7
Cluster 2: Contact with patient 3								
2020 Jan 17–24, 28								
7 (mother)	49 y/F	6	Feb 3	1	28.1	>40.0	32.5	9
8 (sister)	16 y/F	7	Feb 4	0	28.8	>40.0	23.3	9
9 (father)§	50 y/M	NA	Feb 4¶	NA	>40.0	>40.0	>40.0	9
		NA	Feb 11	NA	30.0	34.0	33.0	
		NA	Feb 18	NA	26.0	28.0	30.0	
2020 Jan 22, 28								
10 (cousin)	42 y/F	4	Feb 1	2	29.4	36.0	31.4	4
2020 Jan 28								
11 (neighbor)	55 y/F	3	Jan 31	6	23.0	30.0	28.0	6
2020 Jan 28–Feb 3, contact with patients 10 and 11								
12 (grandchild of patient 10)#	3 mo/M	3	Feb 6	0	30.0	30.9	30.8	8

*Ct, cycle threshold; NA, not applicable because patient was asymptomatic; *RdRp*, RNA-dependent RNA polymerase.  †Possible incubation period calculation was based on last day of possible contact with patients and onset of disease. Cluster 1 travelers had returned from Wuhan, China, on January 17, 2020, on the same flight from Guangzhou, China, to Hanoi, Vietnam. All cluster 2 patients had contact with patient 3 of cluster 1. Although patient 3 returned from the epicenter of the outbreak and is the only patient with a link to cluster 2, the possibility of virus transmission between patients within the same cluster cannot be ruled out. ‡Virus shedding period was the interval from the day on which a sample was positive by real-time PCR to the day on which virus RNA was negative by real-time PCR. Real-time PCR was performed to detect 3 genes of the severe acute respiratory syndrome coronavirus 2 virus; namely, the E, N, and *RdRp* genes. Ct values >40.0 were considered negative. §Patient was virus positive by real-time PCR on 2 consecutively collected samples. ¶Denotes sampling date because this patient was asymptomatic #Patient had no direct contact with persons in cluster 1 but had close contact with persons in cluster 2.

Clinical signs, including fever and cough, were demonstrated by 11 patients an average of 9.9 (± 5.4) days after travel or close contact with patients, indicating that the incubation period was 1–2 weeks after exposure (*4*) (Appendix). In these patients, virus shedding was detected from day 1 after illness onset through day 19 (4.6 days) after potential initial exposure (Table). Of note, 1 patient in cluster 2 (patient 9, a 55-year-old man) was asymptomatic, but virus shedding was detected for up to 9 days (Table). This finding confirms virus shedding in asymptomatic patients and indicates possible transmission during the asymptomatic period. In this context, virus was isolated from 3 patients by inoculation of throat swab samples onto Vero cells. Phylogenetic analyses of an isolate from patient 3 showed that the full-length genome had high sequence homology (99.96%) to a SARS-CoV-2 isolate identified in Wuhan, China (*5*).

Although close-contact transmission of SARS-CoV-2 between family members has been identified (*6*), evidence of virus circulation within the community in Vietnam is limited. We describe 6 cases of close-contact transmission between family members and those living in close proximity and determined virus shedding patterns of 12 patients in Vietnam. Because the virus was not circulating locally, our data provide insight into viral RNA shedding patterns from a potential point of exposure. Although patients were discharged after 2 consecutive negative PCR results, further assessment of the correlation of virus shedding with infectivity will be key for determining the risk for transmission during the viral RNA–positive phase. Given that the possibility of virus transmission between patients within the same cluster could not be ruled out and that the incubation period varies among individuals, uncertainties remain surrounding the estimated incubation period based on contact with patients. Further epidemiologic data are expected to improve the estimates of incubation period. We found limited community transmission of SARS-CoV-2 in Vietnam, and our data indicate that viremic travelers may pose a risk for introduction of virus strains that could potentially lead to outbreaks within a local community.

Appendix Virus shedding patterns detected during study of severe acute respiratory syndrome coronavirus 2 shedding by travelers, Vietnam, 2020.
