# Supplementary material for: Severe Acute Respiratory Syndrome Coronavirus 2 Shedding by Travelers, Vietnam, 2020
Source: Emerg Infect Dis. 2020 Jul;26(7):1624–6. doi: 10.3201/eid2607.200591 (PMC7323563; doi:10.3201/eid2607.200591)
Supplement: Appendix — Virus shedding patterns detected during study of severe acute respiratory syndrome coronavirus 2 shedding by travelers, Vietnam, 2020. [file 20-0591-Techapp-s1.pdf]

Article DOI: https://doi.org/10.3201/eid2607.200591

Severe Acute Respiratory Syndrome Coronavirus 2 Shedding by Travelers, Vietnam, 2020

Appendix

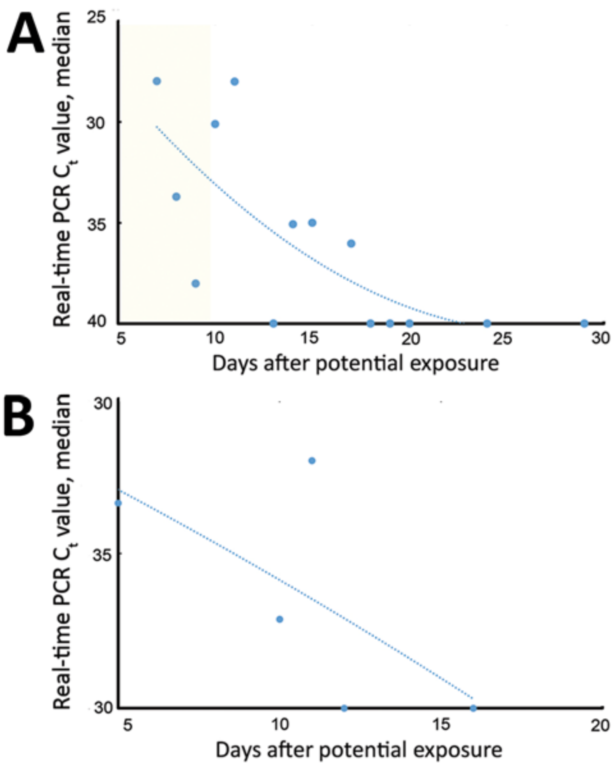

Appendix Figure. (A) Virus shedding patterns as detected in throat-swab samples from SARS-CoV-2 patients and (B) in serial throat-swab samples obtained from patient 2. Values were determined from median Ct values of E gene, N gene, and RdRp gene. Yellow region indicates average days between potential initial exposure and days of disease onset (9.9 ±5.4 days).
